# Embolism of the Aorta

**Published:** 1896-04

**Authors:** Otto Noack

**Affiliations:** Reading, Pa.


### EMBOLISM OF THE AORTA.

By Dr. OTTO NOACK,
READING, PA.

A sorrel mare, belonging to H. K. Getz, was brought to me for examination December 26, 1895. By outward appearance I could not detect any symptoms of disease. The owner stated that whenever the mare was driven, no matter how slowly, she would sweat profusely, walk lame, and then begin to stagger. I then made an examination of the mare while standing. Mucous membrane of nose and eyes very pale, beating of heart feeble, regurgitation of blood into the heart could be heard. The heart-sounds were irregular and scarcely audible; pulse feeble, but could be distinctly felt. I then ordered the horse to be hitched to a buggy to see the different symptoms when driven. The first four blocks the mare trotted all right, showing nothing unusual, then suddenly a peculiar symptom could be seen. The tail got stiff and twisted in a peculiar manner. She ceased trotting and walked very slowly, then ceased altogether. The muscles in the hind-legs were contracted as if in cramps. These symptoms disappeared after a lapse of several minutes, beginning to walk again, sweating profusely, but then the hind-part staggered so severely that I was compelled to halt. Breathing was very difficult and accelerated; head, fore, and middle part of the body were covered with perspiration ; not a drop of sweat on the hind-part could be seen, these symptoms showing that the circulation in the end-part of the aorta and femoral arteries was greatly interrupted.

*Diagnosis:* Heart-failure and embolismus of the aorta. *Prog-*

*nosis:* Very grave. I advised the best would be to kill the mare. Gave her chloroform, and dissected her the same day, December 28, 1895.

*Autopsy:* Upon opening the abdominal cavity nothing abnormal could be seen. After removing the intestines a fibroid tumor the size of a fist was located right in front of the left kidney, hanging on the peritoneum. The kidneys were enlarged, the color being of a very dark brown, and, by cutting, the glomeruli could be recognized. The liver was greatly enlarged, rims swollen, and very dark. The spleen was also enlarged. Upon opening the breast-cavity the smell of chloroform could be noticed. The pericardium contained a serous liquor. The heart was very large and heavy, weighing twenty-three and a half pounds; the heart-muscle was soft and relaxed, also hypertrophied. The left ventricle contained a yellowish gelatinous mass, this being fibrinous. The heart muscle was a pale gray. Then I cut the aorta posterior out, and upon opening the same it contained fibrinous embolus, commencing from the arteriæ renales and filling up the whole end-part of the aorta and femoral arteries, over three inches long and three-quarters of an inch in diameter.

*Anatomical Diagnosis:* Tumor fibrosus in cavitate abdominali, nephritis parenchymatosa, hepatitis, intumescentia lienis, pericarditis, myocarditis et myositis parenchymatosa, dilatatio et hypertrophia cordis, embolismus aortæ posterioris.

